# Implication of Xenobiotic Metabolizing Enzyme gene (CYP2E1, CYP2C19, CYP2D6, mEH and NAT2) Polymorphisms in Breast Carcinoma

**DOI:** 10.1186/1471-2407-8-109

**Published:** 2008-04-18

**Authors:** Achraf Khedhaier, Elham Hassen, Noureddine Bouaouina, Sallouha Gabbouj, Slim Ben Ahmed, Lotfi Chouchane

**Affiliations:** 1 Laboratoire d'Immuno-Oncologie Moleculaire, Faculté de Médecine de Monastir, Tunisia; 2 Department of Cancérologie Radiothérapie CHU Farhat Hached, Sousse, Tunisia; 3 Department of Service de Carcinologie Médicale, CHU Farhat Hached, Sousse, Tunisia; 4 Department of Genetic Medicine, Weill Cornell Medical College in Qatar, Qatar

## Abstract

**Background:**

Xenobiotic Metabolizing Enzymes (XMEs) contribute to the detoxification of numerous cancer therapy-induced products. This study investigated the susceptibility and prognostic implications of the CYP2E1, CYP2C19, CYP2D6, mEH and NAT2 gene polymorphisms in breast carcinoma patients.

**Methods:**

The authors used polymerase chain reaction and restriction enzyme digestion to characterize the variation of the CYP2E1, CYP2C19, CYP2D6, mEH and NAT2 gene in a total of 560 unrelated subjects (246 controls and 314 patients).

**Results:**

The mEH (C/C) mutant and the NAT2 slow acetylator genotypes were significantly associated with breast carcinoma risk (p = 0.02; p = 0.01, respectively). For NAT2 the association was more pronounced among postmenopausal patients (p = 0.006). A significant association was found between CYP2D6 (G/G) wild type and breast carcinoma risk only in postmenopausal patients (p = 0.04). Association studies of genetic markers with the rates of breast carcinoma specific overall survival (OVS) and the disease-free survival (DFS) revealed among all breast carcinoma patients no association to DFS but significant differences in OVS only with the mEH gene polymorphisms (p = 0.02). In addition, the mEH wild genotype showed a significant association with decreased OVS in patients with axillary lymph node-negative patients (p = 0.03) and with decreasesd DFS in patients with axillary lymph node-positive patients (p = 0.001). However, the NAT2 intermediate acetylator genotype was associated with decreased DFS in axillary lymph node-negative patients.

**Conclusion:**

The present study may prove that polymorphisms of some XME genes may predict the onset of breast carcinoma as well as survival after treatment.

## Background

Breast carcinoma is the most frequent malignancy in women [1] and represents the second leading cause of cancer death among women (15% of cancer deaths) [2]. With 200,000 cases in the U.S.A. (27% of all cancers in women) [3]; 320,000 cases in Europe (31% of all cancers in women) [4] and one million new cases diagnosed worldwide every year, breast carcinoma is still a major health problem in many developed countries. In Tunisia, breast carcinoma accounts for 20–25% of malignant tumors in women with an annual incidence of about 800–1000 cases [5]. The etiology of breast carcinoma is still poorly understood in spite of known breast carcinoma risk factors such as age, reproductive events (menarche, menopause, pregnancy, breastfeeding), exogenous hormones (hormone replacement therapy and oral contraceptives), lifestyle and environment risk factors (pollution, alcohol, diet, obesity), ionizing radiation, chemo preventive agents, as well as genetic factors (high-and low penetrance breast cancer susceptibility genes) [6].

In hereditary breast carcinoma, mutations in highly penetrant genes such as BRCA1 or BRCA2 confer a relatively high risk for developing breast carcinoma, though this risk accounts only for about 5 to 10% of all breast carcinoma cases [7]. It is suggested that the effect of low penetrance cancer susceptibility genes modulated by environmental exposure and lifestyle factors are likely to account for most of sporadic breast carcinoma cases [8]. In the latters, the proportion of breast carcinoma attributable to such genetic traits, in combination with environmental exposure, is likely to be much higher than the hereditary proportion and accounts for 90 to 95% of all breast carcinoma cases [8].

Candidate genes of low-penetrance breast carcinoma susceptibility include those encoding for Xenobiotic Metabolizing Enzymes (XMEs) involved in carcinogen metabolism and detoxification [9]. These XMEs can be divided into phase I enzymes (Cytochrome P450 family: CYP2E1, CYP2C19, CYP2D6 and mEH) that metabolically activate potentially carcinogenic forms and phase II enzymes (N-acetyl- and gluthatione-S-transferases families: NAT1, NAT2, GSTT1, GSTM1 and GSTP1) that metabolically inactivate carcinogens to increase its solubility in such a way as to facilitate its excretion and detoxification [10]. XMEs are also involved in the metabolism of a wide range of drugs including a variety of anticancer chemotherapy agents. These agents exert their anti-neoplastic effects by generating reactive oxygen species (ROS) whose direct cytotoxic effects are in many cases the proximate cause of tumor cell death and are likely to have initial and immediate impact on treatment efficacy [11]. Furthermore, altered intratumoral genes coding for XMEs were suggested as a potential molecular mechanism to explain metabolism's alteration of the chemotherapy agents and consequently the reduced treatment efficacy and tumor resistance [12].

Polymorphisms in both phase I and phase II enzyme genes may result in alteration of their expression, function and activity. Several studies have attempted to tackle the genetic polymorphisms of different XMEs alone or in combinations with altered risks of breast carcinoma [9,10]. Moreover, because of the great number of carcinogen-activating and detoxifying enzymes, the complexity of exposures to environmental carcinogens and gene-gene interactions, evaluating a single polymorphic enzyme may not be sufficient to assess their role in carcinogenesis. However, accumulating series of alleles "at risk" considerably increase the cancer risk.

The present study first investigated the relationship between DNA variants in Cytochrome P 450 (CYP) -2E1, -2C19, -2D6, microsomal Epoxide Hydrolase (mEH) and N-acetyltransferase -2 (NAT2) enzymes and susceptibility to breast carcinoma. Then the potential contribution of the combined multilocus genotypes in breast carcinoma susceptibility was examined. Finally, the study tried to establish a potential association of these gene variations with tumor clinical-pathological characteristics, with survival and relapse after treatment from breast carcinoma.

## Methods

### Subjects

A total of 560 unrelated subjects (246 controls and 314 patients), living in Sousse on the middle coast of Tunisia, were enrolled in this study. Clinical data about the cohort of the 314 patients recruited from the department of Radiation Oncology and Medical Oncology of Sousse Hospital, were collected between 1994 and 2002. All patients included in this study had primary breast carcinoma, with unilateral breast tumors. The patients had a mean age of 52 ± 24 years. The median follow-up was 36 months (range, 1 to 120 months). At the time of analysis, 76 patients relapsed (local or distant recurrence). Among them, 36 patients died from breast carcinoma (47.3%). A detailed description of the clinical pathological characteristics of this cohort was summarized in Table 1 and reported elsewhere [5,13]. Control subjects having a mean age of 41 ± 14 years, were healthy blood donors having no evidence of any personal or family history of cancer or other illnesses. Written informed consent was obtained from all subjects.

**Table 1:** Clinical features of breast carcinoma patients

**Variables**	**Pourcentage (%)**
*Clinical tumor size*	
T_1_–T_2_	62.28
T_3_–T_4_	37.72
*Lymph node status*	
N(-)	54.2
N(+)	69.12
*SBR grading*	
1 – 2	80.88
3	86.76

### Genomic DNA extraction

Genomic DNA was extracted from peripheral blood leukocytes by a salting procedure [14]. Briefly, 10 ml of blood were mixed with triton lysis buffer (0.32 M sucrose, 1% Triton X-100, 5 mM MgCl_2_, H_2_O, 10 mM Tris-HCl, pH 7.5). Leukocytes were spun down and washed with H_2_O. The pellet was incubated with proteinase K at 56°C and subsequently salted out at 4°C using a concentrated NaCl solution. Precipitated proteins were removed by centrifugation. The DNA in supernatant fluid was precipitated with ethanol. The DNA pellet was dissolved in 400 μl of sterile distilled water.

### Polymorphism analysis

Polymorphic sites of the mEH (T348C), CYP2E1 (C-1091T), CYP2C19 (intron 4/exon 5 G-A splice site mutation), CYP2D6 (G1934A), NAT2 (C481T, G590A, A803G and G857A) genes were genotyped by Polymerase Chain Reaction-Restriction Fragment Length Polymorphism (PCR-RFLP) assay. Previously reported primers and restricted enzymes in RFLP-PCR are listed in Table 2. All PCR reactions were performed in an independent blinded duplicate manner and for each polymorphism some samples were confirmed by sequencing the PCR products (Abi Prism 310, Applied Biosystems).

**Table 2:** Primers and restriction enzymes used for polymorphism genotyping.

**Genes**	**Polymorphisms**	**Primers**	**Restriction enzymes**	**References**
**mEH**	T348C	Sens : 5' CTT GAG CTC TGT CCT TCC CAT CCC 3'	Tth 111I	15
		Antisens : 5' AAT CTT AGT CTT GAA GTG ACG GT 3'		
**CYP2E1**	C-1091T	Sens : 5' CCA GTC GAG TCT ACA TTG TCA 3'	PstI	15
		Antisens : 5' TTC ATT CTG TCT TCT AAC TGG 3'		
**CYP2C19**	Intron4/exon5 G-A splice mutation	Sens : 5' AAT TAC AAC CAG AGC TTG GC 3'	SmaI	16
		Antisens : 5' TAT CAC TTT CCA TAA AAG CAA G 3'		
**CYP2D6**	G1934A	Sens : 5' GCT TCG CCA ACC ACT CCG 3'	BstN1	17
		Antisens: 5' AAA TCC TGC TCT TCC GAG GC 3'		
**NAT2**	C481T, G590A, A803G, G857A	Sens : 5' GCT GGG TCT GGA AGC TCC TC 3'	KpnI, TaqI, DdeI, BamHI	17
		Antisens : 5' TTG GGT GAT ACA TAC ACA AGG G 3'		

The polymorphic site of the mEH (T348C) and CYP2E1 (C-1091T) genes was simultaneously revealed by Multiplex Polymerase Chain Reaction (PCR)-Restriction Fragment Length Polymorphism (RFLP) assay described by Salama et al. with a slight modification [15]. A multiplex PCR reaction was performed in a total volume of 50 μl containing 100 ng of genomic DNA, 200 μmol dNTPs, 2 mM MgCl_2_, 1 × Taq polymerase buffer, 100 pmol of CYP2E1 (X3, X4) and mEH (X5, X6) primers and 1 U of Taq DNA polymerase (Amersham, Paris, France). The reaction conditions used with the thermal cycler (Biometra, Göttingem, Germany) were as follows: the initial incubation at 94°C for 5 min, followed by 35 cycles of incubation at 94°C for 2 min; 59°C for 1 min, and 72°C for 1 min and achieved by a final incubation at 72°C for 10 min. To verify proper amplification conditions, 10 μl of PCR product were analyzed on a 2% agarose gel and stained with ethidium bromide, the amplification of CYP2E1 and mEH was revealed by the presence of bands at 410 and 231 bp, respectively. To detect CYP2E1 (C-1091T) and mEH (T348C) polymorphisms, amplified DNA was digested with 10 U of PstI (37°C, 3 h) and Tth 111I (65°C, 3 h) endonucleases respectively. CYP2E1 wild type allele was characterized by the absence of PstI restriction site and revealed by a band at 410 bp. The mutant allele was characterized by the presence of PstI restriction site and revealed by a band at 290 bp and a band at 120 bp. Tth 111I digestion produced 209 and 22 bp size fragments for mEH mutant allele, whereas the mEH wild type allele remained undigested (231 bp).

The splice-site mutation of CYP2C19 (G-A, exon 5) was analyzed by PCR-RFLP assay described by De Morais et al. with some modifications [16]. PCR incubation was performed in a total volume of 20 μl containing 200 ng of genomic DNA, 200 μmol dNTPs, 1.5 mM MgCl_2_, 1 × Taq polymerase buffer, 0.25 μmol of CYP2C19 (X1, X2) primers and 1 U of Taq DNA polymerase. Amplification was done with one pretreatment cycle at 94°C for 4 min, 55°C for 2 min and 72°C for 1 min, followed by 30 cycles with 0.5 min denaturation (94°C), 1 min annealing (55°C) and 1.5 min elongation (72°C), achieved by a 5 min final elongation (72°C). This yielded a 169 bp fragment. Restriction enzyme cleavage was performed overnight on 10 μl of PCR product after addition of 10 U of SmaI restriction enzyme. The SmaI digestion produced fragments of 120 and 49 bp for CYP2C19 wild type allele; whereas the mutant allele remains undigested (169 bp).

A PCR-RFLP method described by Lemos et al. with a slight modification was used for the detection of the CYP2D6 G1934A mutation [17]. Amplification was carried out in a 20 μl reaction volume containing 200 ng of genomic DNA, 200 μmol dNTPs, 1.5 mM MgCl_2_, 1 × Taq polymerase buffer, 150 ng of each primer (Pc1, Pc2) and 1 U of Taq DNA polymerase. A 334 bp fragment was amplified after an initial denaturation at 94°C for 5 min and 30 cycles with 1 min denaturation (94°C), 1 min annealing (60°C) and 2 min elongation (72°C), followed by 5 min final elongation (72°C). Digestion of 10 μl of the PCR product was carried out with 5 U of the restriction enzyme BstN1 (60°C, 1 h). CYP2D6 wild-type allele was identified by the presence of 230 and 104 bp size fragments. CYP2D6 mutant allele did not have a BstN1 restriction site and remained undigested (334 bp).

The four most common NAT2 single nucleotide polymorphisms (C481T, G590A, A803G and G857A) associated with low NAT2 activity were genotyped using a modified version of PCR-RFLP described by Lemos et al. [17]. Essentially as published, aliquots of 100 ng of genomic DNA were amplified in a 60 μl reaction volume containing 400 μmol dNTPs, 1.5 mM MgCl_2_, 1 × Taq polymerase buffer, 100 ng of each primer (Pn1, Pn2) and 1 U of Taq DNA polymerase. After an initial denaturation at 94°C for 5 min, 34 cycles were performed consisting of denaturation at 94°C for 0.5 min, annealing at 60°C for 0.5 min and elongation at 72°C for 0.75 min, completed with a final cycle of 60°C for 5 min and 72°C for 5 min. This resulted in the amplification of a 540 bp fragment. Subsequently, 10 μl of this reaction were then subjected to restriction enzyme analysis with 5 U of KpnI, TaqI, DdeI and BamHI for the detection of mutations C481, G590A, A803G and G857A, respectively. The wild-type allele (NAT 2 *4) was identified by complete digestion by KpnI, TaqI and BamHI, but not DdeI. The mutations either destroyed the recognition sites of the first three enzymes or created a new one for the fourth. NAT 2 *5A, *6B, *7A and *12A alleles were identified by the presence of C481T, G590A, G857A and A803G mutations respectively. The NAT 2 *5B allele was identified by the presence of both C481T and A803G mutations. Restriction enzymes were obtained from New England BioLabs. Digestion conditions were performed according to the manufacturer's instructions and summarized in Table 3. Digestion products were separated at the appropriate concentrations on a 2, 3 or 4% Low-melting point agarose gel and stained with ethidium bromide.

**Table 3:** Restriction enzyme conditions used for NAT2 polymorphism genotyping.

**Polymorphisms**	**Restriction Enzymes**	**Temperature and incubation time**	**Fragment size (pb)**	**Agarose (%)**
**C481T**	KpnI (5 U)	37°C – 4 h 30	114 + 426	2
**G590A**	TaqI (5 U)*	65°C – 1 h 30	223 + 170 + 147	3
**A803G**	DdeI (5 U)**	37°C – 4 h 30	65 + 346 + 25 + 104	4
**G857A**	BamHI (5 U)	37°C – 4 h 30	490 + 50	2

* two sites; ** three sites of restriction enzymes

### Statistical analysis

The genotype frequencies of different genes were tested for Hardy-Weinberg equilibrium for both patient and control groups using the χ^2 ^test. The same test was used to evaluate significant associations between the disease (breast carcinoma *versus *controls) and different genotypes. The differences were considered significant if the P-value did not exceed 0.05. Odd ratios (ORs) and 95% confidence intervals (CIs) were calculated by unconditional logistic regression. When expected values in contingency tables were under 5, Fisher's exact test was used.

The clinical pathological parameters studied herein were age, nodal status, SBR (Scraff, Bloom and Richardson) tumor grade, clinical tumor size and response to treatment. The clinical pathological parameters were dichotomised as follows: Age (<50 years *versus *≥ 50 years), nodal status (≥ 1 *versus *no positive lymph node), SBR tumor grade (1–2 *versus *3), clinical tumor size (T_1_–T_2 _versus T_3_–T_4_).

Disease-free survival (DFS) was defined as the time from the date of diagnosis to the first local or distant recurrence or to the last contact. Breast carcinoma-specific overall survival (OVS) was defined as the time from the date of diagnosis to death if the patient died from breast carcinoma or to the last contact. Six-year survival rates were estimated, and survival curves were plotted according to Kaplan and Meier. Differences between groups were calculated by the log-rank test.

Statistics were performed using SEM-STATISTIQUES software (Centre Jean Perrin, Clermont-Ferrand, France).

## Results

### XMEs gene polymorphisms and susceptibility to breast carcinoma

The number of polymorphism-genotyped individuals was dependent upon DNA availability. All genotype distributions did not diverge significantly from Hardy-Weinberg equilibrium for both patient and control groups separately. There were no significant differences between patients and controls in the genotype frequencies for CYP2C19, CYP2E1 and CYP2D6 genes (Table 4). When the patients were stratified according to their menopause status, the CYP2D6 (G/G) wild genotype frequency was found to be significantly higher in postmenopausal patients than in controls (OR = 1.79; p = 0.04), suggesting an association between the homozygous CYP2D6 wild genotype and the late onset of breast carcinoma. However, the heterozygous CYP2D6 (G/A) genotype was associated with a protective effect against breast carcinoma (OR = 0.5; p = 0.02) in postmenopausal patients. A statistically significant association was found between mEH mutant homozygous genotype (C/C) and breast carcinoma in Tunisians (OR = 2.06; p = 0.02). This association seems to be particularly higher in premenopausal patients (OR = 2.3; p = 0.01).

**Table 4:** CYP2E1, CYP2C19, CYP2D6, mEH and NAT2 genotype frequencies in control subjects and in patients with breast carcinoma.

**Genotypes**	**Controls**		**All Patients**				**Premenopausal Patients**				**Postmenopausal Patients**			
	n	*f*	n	*f*	P	OR (95%CI)	n	*f*	p	OR (95%CI)	n	*f*	p	OR (95%CI)
**CYP2E1 **(C-1091T)	244		304				192				111			
CYP2E1 (C/C)	232	0.95	296	0.97	N.S	1.91(0.72–5.22)	186	0.97	N.S	1.6(0.55–4.89)	109	0.98	N.S	2.82(0.58–18.57)
CYP2E1 (C/T)	12	0.05	08	0.03	N.S	0.52(0.19–1.4)	06	0.03	N.S	0.62(0.2–1.83)	02	0.02	N.S	0.35(0.05–1.71)
CYP2E1 (T/T)	00	0.00	00	0.00	N.S	-	00	0.00	N.S	-	00	0.00	N.S	-

**CYP2C19 **(exon5 G-A)	240		304				193				109			
CYP2C19 (G/G)	197	0.822	239	0.787	N.S	0.8(0.51–1.26)	155	0.804	N.S	0.89(0.53–1.49)	83	0.762	N.S	0.7(0.39–1.25)
CYP2C19 (A/G)	41	0.170	61	0.200	N.S	1.22(0.77–1.93)	37	0.191	N.S	1.15(0.68–1.94)	23	0.211	N.S	1.30(0.71–2.38)
CYP2C19 (A/A)	02	0.008	04	0.013	N.S	1.59(0.25–12.56)	01	0.005	N.S	0.62(0.02–8.76)	03	0.027	N.S	3.37(0.45–29.24)

**CYP2D6 **(G1934A)	230		300				189				109			
CYP2D6 (G/G)	167	0.726	235	0.783	N.S	1.36(0.9–2.07)	145	0.767	N.S	1.24(0.78–1.99)	90	0.827	**0.04**	1.79(0.97–3.31)
CYP2D6 (G/A)	56	0.243	58	0.193	N.S	0.74(0.48–1.15)	41	0.217	N.S	0.86(0.53–1.42)	15	0.137	**0.02**	0.5(0.25–0.96)
CYP2D6 (A/A)	07	0.031	07	0.023	N.S	0.76(0.24–2.48)	03	0.016	N.S	0.51(0.1–2.24)	04	0.036	N.S	1.21(0.29–4.74)

**mEH **(T348C)	244		306				194				110			
mEH1 (Tyr/Tyr)	113	0.463	149	0.486	N.S	1.1(0.77–1.56)	97	0.500	N.S	1.16(0.78–1.72)	52	0.472	N.S	1.04(0.65–1.67)
mEH2 (Tyr/His)	115	0.471	119	0.388	N.S	0.71(0.5–1.02)	70	0.360	**0.02**	0.63(0.42–0.95)	47	0.427	N.S	0.84(0.52–1.35)
mEH3 (His/His)	16	0.065	38	0.124	**0.02**	2.02(1.06–3.89)	27	0.139	**0.01**	2.3(1.15–4.64)	11	0.1	N.S	1.58(0.66–3.77)

**NAT2**	237		290				184				105			
Rapid Acetylators	14	0.059	24	0.082	N.S	1.44(0.69–3)	18	0.097	N.S	1.73(0.79–3.79)	06	0.057	N.S	0.97(0.32–2.79)
Intermediate Acetylators	105	0.443	92	0.317	N.S	0.58(0.4–0.85)	61	0.331	N.S	0.62(0.41–0.95)	30	0.285	**0.006**	0.52(0.3–0.85)
Slow Acetylators	118	0.497	174	0.600	**0.01**	1.51(1.05–2.17)	105	0.570	N.S	1.34(0.89–2.01)	69	0.657	**0.006**	1.93(1.17–3.2)

The chi-square test was used to determine whether significant differences (p value) were observed when the patient group was compared with the control group. f, frequencies; NS: not significant.

Genotyping analysis of NAT2 polymorphisms revealed 17 different genotypes resulting from the combinations of the following 6 alleles: NAT 2 *4 (wild-type), *5A, *5B, *6B, *7A and *12A (mutants). Only NAT2*4 and *12A alleles represented rapid acetylators while the other alleles represented slow ones (Table 3). Among the 17 genotypes, the heterozygous slow acetylators NAT2*5A/*6B were found to be statistically significant as a risk factor for breast carcinoma (Table 5). Heterozygous NAT2*12A/*5B genotype (intermediate acetylator) was significantly more frequent in controls than in patients (8.86% vs 2.41%) indicating a protective effect of this gene variant against breast carcinoma (OR = 0.25, p = 0.001). When the 17 genotypes were pooled according to their acetylator status, the only significant difference was in the frequency of NAT2 slow acetylator genotype, which was higher in patients than in controls (60% vs 49.7%). Thus, NAT2 slow acetylator genotype was significantly associated with breast carcinoma risk (Table 4). This association was more pronounced among postmenopausal patients (OR = 1.93, p = 0.006). However, a significant association with a protective effect in postmenopausal patients can be assigned to NAT2 intermediate acetylator genotype (OR = 0.52, p = 0.006).

**Table 5:** NAT2 Genotype Frequencies in Control Subjects and in Patients with Breast Carcinoma.

**NAT2 genotypes**	**Controls (n = 237)**	**All Patients (n = 290)**	**p**	**OR (95%CI)**
	n	n		
NAT2*4/4	13	23	NS	1.48(0.7–3.18)
NAT2*4/12A	01	00	NS*	-
NAT2*12A/12A	00	01	NS*	-
NAT2*4/5A	01	07	NS*	-
NAT2*4/5B	41	45	NS	0.88(0.54–1.43)
NAT2*4/6B	32	27	NS	0.66(0.37–1.17)
NAT2*4/7A	05	04	NS*	-
NAT2*12A/5B	21	07	**0.001**	0.25(0.1–0.65)
NAT2*12A/6B	05	02	NS*	-
NAT2*5A/5B	02	04	NS*	-
NAT2*5A/6B	00	05	0.04*	-
NAT2*5A/7A	00	01	NS*	-
NAT2*5B/5B	35	53	NS	1.29(0.79–2.11)
NAT2*5B/6B	54	68	NS	1.04(0.68–1.59)
NAT2*5B/7A	06	11	NS	1.52(0.51–4.69)
NAT2*6B/6B	15	25	NS	1.40(0.69–2.86)
NAT2*6B/7A	06	07	NS	0.95(0.28–3.24)

The chi-square test was used to determine whether significant differences (p value) were observed when the patient group was compared with the control group. NS: not significant; *: Fisher test was used.

A combined genotype analysis revealed that mEH (C/C)-NAT2 slow acetylator genotype combinations was associated with breast carcinoma risk (OR = 2.18; p = 0.04). The distribution of this genotype combination was 3.79% in controls and 7.93% in patients. Conversely, the mEH (C/T)-NAT2 (Rapid/Slow acetylators) combination was highly associated with a protective effect against breast carcinoma (OR = 0.44; p = 0.0004).

### Survival analysis and prognostic significance of XME gene polymorphisms

**Figure 1:**
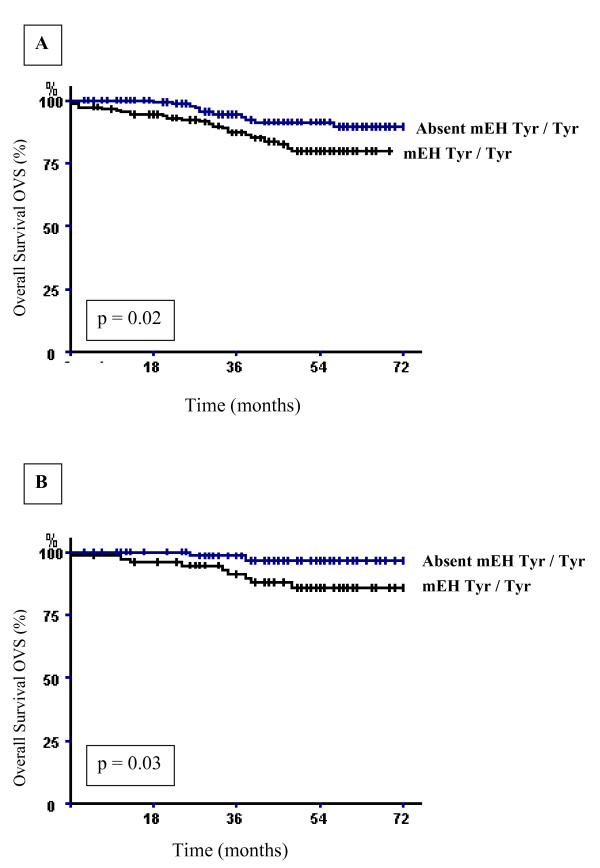
**Breast carcinoma-specific overall survival (OVS) of breast carcinoma patients according to the presence or absence of the mEH wild type genotype; (A) among the whole breast carcinoma patients and (B) among axillary's lymph node-negative ones.** p denotes the log-rank test value.

When the relationship between the distribution of CYP2E1, CYP2C19, CYP2D6 and NAT2 genotypes in all patients and the survival (OVS or DFS) was tested, no significant differences were observed between the different Kaplan-Meier survival curves. However, mEH wild homozygous genotype (Tyr/Tyr) was significantly associated with decreased breast carcinoma specific overall survival (Fig. 1) but not Disease-free survival. The estimated 6-year OVS rate in the groups of patients carrying or not the mEH wild homozygous genotype (Tyr/Tyr) was 79.41% and 89.7%, respectively (log-rank test, p = 0.02).

**Figure 2:**
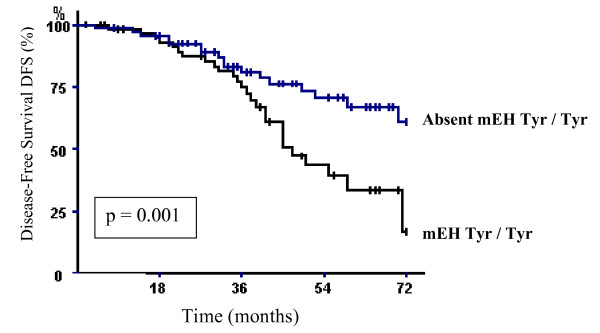
**Breast carcinoma-specific disease-free survival (DFS) of axillary's lymph node-positive breast carcinoma patients according to the presence or absence of the mEH wild type genotype.** p denotes the log-rank test value.

Further analyses were conducted to explore whether the studied gene polymorphisms were associated with survival among different subgroups of patients (Age, nodal status, SBR tumor grade, clinical tumor size). The only significant associations were found with axillary lymph node-negative or -positive patients. Indeed, the overall survival was significantly shorter in axillary lymph node-negative patients carrying the mEH wild homozygous genotype (Tyr/Tyr). The 6-year OVS rate in the groups of patients carrying or not mEH wild genotype was 85.29% and 97.05%, respectively (log-rank test, p = 0.03) (Fig. 1). In addition, statistical significant differences in DFS were seen between patients axillary lymph node-positive carrying the mEH wild homozygous genotype (Tyr/Tyr) and those without (log-rank test, p = 0.001). The group of patients with mEH wild homozygous genotype (Tyr/Tyr) had lower breast carcinoma disease free-survival than those without mEH wild genotype. The 6-year DFS rates were 16.2% and 60.3%, respectively (Fig. 2).

**Figure 3:**
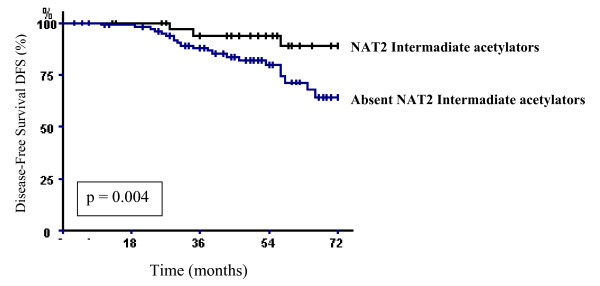
**Breast carcinoma-specific disease-free survival (DFS) of axillary's lymph node-negative breast carcinoma patients according to the presence or absence of the NAT2 intermediate acetylator genotype.** p denotes the log-rank test value.

Still among the axillary lymph node-negative patients, when a DFS comparison was made between patients who had NAT2 intermediate acetylator genotype and those who did not, an increase in DFS was observed in patients carrying NAT2 intermediate acetylator genotype (log-rank test, p = 0.04). The 6-year DFS rate in the group of patients with NAT2 intermediate acetylator genotype was 89.7% and 63.3% in patients without this genotype (Fig. 3).

There were no statistically significant associations between all the five gene polymorphisms and the rest of clinical pathological parameters such as clinical response to chemotherapy, tumor grade and clinical tumor size analyzed in our study.

## Discussion

The CYP2D6 polymorphism analysis revealed that the homozygous wild type genotype increased susceptibility to breast carcinoma in the selected population of postmenopausal patients, however controversial results were reported. Indeed, De Jong et al. found that homozygous mutant CYP2D6 genotype increased the risk of breast carcinoma [18]. In the selected population, the heterozygous CYP2D6 genotype was also found to be associated to a protective effect against breast carcinoma. Conversely, Ladona et al. reported a significant association between the heterozygous CYP2D6 genotype and breast carcinoma risk among postmenopausal patients [19]. More recently, an association with a protective effect against papillary thyroid cancer has been found with the homozygous mutant CYP2D6 genotype and similar results have been reported for tumors at other sites, such as lung cancer and leukaemia [17,20,21]. The CYP450 enzymes, including CYP2D6, are responsible for the activation of procarcinogens and genotoxic metabolites [22]. The CYP2D6 polymorphism G1934A leads to a disruption of the reading frame and a truncated non functional protein [23,24]. Therefore, individuals with heterozygous or mutant homozygous CYP2D6 genotypes have poor or no enzyme activity respectively. This decreases the formation of genotoxic metabolites and reduces the onset of breast carcinoma development. However, individuals carrying two copies of CYP2D6 wild type allele have higher enzyme activity than those having one or no copy of the wild type allele. Wild homozygous CYP2D6 genotype will metabolize more carcinogens to their genotoxic metabolites [24]. Thus, this higher enzyme activity probably increases DNA damage levels and consequently the risk of breast carcinoma.

Regarding hormone-associated tumors such as ovarian, cervical or prostate cancers, many conflicting data have been found but without conclusive links to CYP450 genes. In this field, numerous studies conducted on different populations and ethnic groups have indicated the absence of a major impact of the CYP2D6, CYP2C19 and CYP2E1 genes in cancer risk [25]. These previous findings are in accordance with ours which demonstrate the absence of any association between CYP2C19 and CYP2E1 gene polymorphisms with breast cancer. In addition, when compared with the allele frequencies of the CYP2D6, CYP2C19 and the CYP2E1 genes in Italian, Portuguese and Egyptian populations, we found similar frequencies in healthy Tunisian individuals [20,26-28]. However, the previous findings can not be generalized even with similarities between healthy individuals. Indeed, it was found in the Portuguese population that the CYP2E1 gene could be associated with prostate cancer risk [29]. Therefore, the study of candidate gene associations to cancer susceptibility has been made difficult due to the different ethnic origins and lifestyle of the study groups. This is particularly true in the case of CYP450 enzyme genes which may influence the carcinogenesis pathway and where environmental factors are relevant and make such comparisons more difficult. Further studies are likely to detect much stronger associations of cancer susceptibility with CYP450 genes taking into account ethnicity and different environmental risk factors.

The current study suggests a significant association between the mEH homozygous mutant genotype and the risk of breast carcinoma, especially, among the subgroup of premenopausal patients. The heterozygous mEH genotype was also found to be protective against breast carcinoma in the selected population. The genetic polymorphism T348C of mEH leads to a substitution of Tyrosine (Tyr) residue 113 by Histidine (His) that is known to decrease the enzyme activity [30]. Thus, "His113" poor activity genotype was responsible for deficient detoxification, accumulation of genotoxic metabolites and probably breast carcinoma initiation. In contrast, the presence of at least one "Tyr113" high activity allele was sufficient to detoxify genotoxic metabolites, thereby protecting breast cells against DNA damage and breast carcinoma risk. Nevertheless, several studies have indicated that the high activity Tyr113 genotype has been associated with increased risk for lung and upper aero-digestive tract cancer [31,32]. In addition, studies conducted in relation to ovarian cancer provide evidence against substantial increased risk associated with mEH polymorphisms [33].

For NAT2, the alleles NAT2*5A and B, NAT2*6B and NAT2*7A observed in our study and respectively corresponding to the Ile114Thr, Arg197Gln and Gly286Glu substitutions, were associated with slow phenotype [34]. However, the allele NAT2*12A resulting from Lys268Arg substitution produced an enzyme with an acetylating capacity similar to the wild type rapid acetylator NAT2*4 allele [34]. Furthermore, individuals carrying one copy of rapid acetylator allele and one copy of slow acetylator allele had an intermediate phenotype [34]. The NAT2 enzyme has been detected in human breast cells. Several studies have indicated that the level of DNA adducts in breast tissues is higher among individuals with the slow NAT2 acetylator genotype, suggesting that slow NAT2 acetylators are more susceptible to adduct-induced DNA damage, which may subsequently contribute to higher risk of breast carcinoma [35]. These findings reinforce our results indicating that individuals who are slow acetylator have increased risk of breast carcinoma. This association was more pronounced among postmenopausal patients. Indeed, slow acetylators have a reduced rate of metabolism of aromatic and heterocyclic amines, and presumably in these individuals, arylamines shift towards the hydroxylation pathway which forms DNA reactive metabolites [35]. Since slow acetylators may be at higher risk of adduct-induced DNA damage, they have an increased susceptibility to breast carcinogenesis. Unlikely, Alberg et al. reported that NAT2 rapid acetylator genotype was associated with increased breast carcinoma risk especially among postmenopausal patients [35], indicating that NAT2 was acting more as an activator of procarcinogens than as a detoxifier [35]. This association should be carefully viewed given the very small number of homozygous NAT2 rapid acetylators and the association has not been seen in other studies. For instance, in Taiwanese breast cancer cases it was found that breast cancer risk was not significantly influenced by NAT2 polymorphisms [36]. In addition, studies which attempted to investigate the NAT2 genetic polymorphisms as an independent risk marker for breast carcinoma have failed [37]. Like CYP450 genes, association studies of NAT2 gene polymorphisms to other hormone-associated tumors are inconsistent. NAT2 may have conflicting associating results for cancer risk in the same population. In fact, slow acetylator genotype was found to have protective and susceptibility effects against prostate and cervical cancer respectively [38,39].

The discrepancy between results, including ours, which tried to address the role of XME gene polymorphisms as risk factors in cancers, particularly breast carcinoma, may be due to several confounding factors including differences in ethnicity of the analyzed populations, sample size, the type of environmental carcinogens to which different population are exposed and the simultaneous involvement of a large number of XME variants.

In this study, mEH gene polymorphism was also associated with the early onset of breast carcinoma whereas polymorphisms in NAT2 and CYP2D6 genes are also correlated with late onset of breast carcinoma. These findings indicate that the penetrance of XME genes varies according to the time of onset of breast carcinoma. This suggests that there could be some new contributing factors, most likely environmental, affecting the penetrance and phenotype expression of XME gene polymorphisms. Indeed, this influence was mostly proposed in certain studies, especially those conducted on hereditary breast carcinoma related to BRCA1/2 genes [40]. This partly explains the emergence of genes such as XMEs but not others at a given epoch in the etiology of breast carcinoma.

For further comprehension of the role played by XME genes polymorphisms in breast carcinoma prognosis and survival, we examined whether the five gene polymorphisms were associated with clinical pathological parameters, Overall survival (OVS) and Disease free Survival (DFS) after treatment. None of the clinical pathological parameters analyzed showed any statistically significant differences by the genotypes of each of the five genes. This is in accordance with results found by Chacko et al. for other XME genes [10] (CYP1A1, GSTT1 and GSTM1) but our results disagree with the findings of Han et al. which showed that polymorphisms of XMEs genes such as CYP1A1, CYP19 and CYP1B1 can influence clinical pathological features of breast carcinoma [9]. The influence of XMEs genes polymorphisms on breast carcinoma prognosis is still poorly understood.

XME enzymes play a crucial role in the detoxification of numerous products induced by cancer therapy [4] and altered intra-tumoral gene coding for XMEs was suggested as a potential molecular mechanism to explain reduced treatment efficacy, tumor resistance [12], and consequently survival and relapse after treatment. Several studies highlighted the role of XME gene polymorphisms such as CYP1A1, CYP1B1, CYP2D6, mEH, GSTT1, GSTM1 and NAT1 in treatment efficacy as well as survival after treatment of breast carcinoma [10,12,13,31,41]. Previous studies were largely carried out on small or heterogeneous populations. In our previous data, the effect of GSTT1 and GSTM1 gene deletions on survival after treatment of breast carcinoma was not evident in the entire population [13]. In the present study, mEH wild-type genotype was significantly associated with decreased OVS in the same Tunisian population of breast carcinoma patients. In addition, the selection of axillary lymph node-positive breast carcinoma allowed the appearance of a significant association between decreased disease-free survival of breast carcinoma and the mEH wild genotype. These results were reinforced by the study of Fritz et al. indicating that mEH was identified as a predictor of the tamoxifen (anticancer drug) response in breast carcinoma [41]. Indeed, individuals carrying mEH wild-type genotype actively detoxify anticancer agents, reduce treatment efficacy and consequently decrease survival after treatment. No association was found with the four other gene polymorphisms. However, there was an increase in DFS for patients with axillary lymph node-negative carrying NAT2 intermediate acetylator genotype.

In conclusion, this study suggests that CYP2D6, mEH and NAT2 gene polymorphisms may be attractive susceptibility markers for breast carcinoma. A further interesting finding is that mEH and NAT2 gene polymorphisms represent a prognostic variable for predicting survival and relapse after treatment in breast carcinoma patients.

## Competing interests

The author(s) declare that they have no competing interests.

## Authors' contributions

AK conceived of the study, conducted data analysis, drafted the manuscript and with SG and EH carried out the experiments. EH contributed to reviewing the manuscript. NB and S-BA provided samples and clinical information. LC conceived, designed and participated in the data analysis and interpretation of the study. All authors read and approved the final manuscript.

## Pre-publication history

The pre-publication history for this paper can be accessed here:


